# Comparative Study of the Pathological Effects of Western Equine Encephalomyelitis Virus in Four Strains of *Culex tarsalis* Coquillett (Diptera: Culicidae)

**DOI:** 10.3389/fpubh.2014.00184

**Published:** 2014-10-09

**Authors:** Marco V. Neira, Farida Mahmood, William K. Reisen, Calvin B. L. James, William S. Romoser

**Affiliations:** ^1^Center for Infectious Disease Research, College of Exact and Natural Sciences, Pontificia Universidad Católica del Ecuador, Quito, Ecuador; ^2^Department of Biomedical Sciences, Tropical Disease Institute, College of Osteopathic Medicine, Ohio University, Athens, OH, USA; ^3^Environmental Health and Engineering, United States Army Public Health Command Region-South, Houston, TX, USA; ^4^Center for Vector-borne Diseases, School of Veterinary Medicine, University of California, Davis, CA, USA

**Keywords:** arbovirus, *Culex tarsalis*, mosquito, pathology, vector competence, western equine encephalomyelitis

## Abstract

Early reports suggested that mosquito cells infected with arboviruses remain viable and undamaged. However, more recent experimental evidence suggests that arboviral infection of mosquito tissues might indeed result in pathological changes, with potential implications for vector survival and virus transmission. Here, we compare the pathological effects of western equine encephalomyelitis virus (WEEV) infection in four strains of *Culex tarsalis* previously reported to differ in their competence as WEEV vectors. Pathological effects were observed in cells of the midgut epithelium, salivary glands, and eggs. Cell rounding and sloughing of midgut epithelial cells was associated with those strains reported to be the least susceptible to WEEV infection, whereas midgut necrosis and vacuolation upon infection were associated with strains showing higher susceptibility. Although pathological effects were sporadically observed in infected salivary glands, further studies are required to evaluate their impact on vector competence. Additionally, the potential implications of observed *C. tarsalis* egg infection with WEEV are discussed.

## Introduction

Transmission of a mosquito-borne virus to a vertebrate host requires mosquito ingestion of a viremic blood meal, subsequent infection of the mosquito’s midgut cells, spread to tissues within the hemocoel, and finally infection of the salivary glands. Following completion of this “extrinsic incubation period,” virions must be released into the saliva and injected into a new host during a subsequent blood meal. Previous investigations have described benign, non-pathological, and chronic viral infections resulting in continuous virus production throughout the lives of infected mosquitoes (1–3). However, pathological effects have also been observed both *in vivo* (4–10) and *in vitro* (11). Weaver et al. (6) reported pathological changes, including cell sloughing and tissue necrosis in *Culex tarsalis* that fed on viremic blood meals containing western equine encephalomyelitis virus (WEEV; Togaviridae, *Alphavirus*). Furthermore, it has been proposed that the effects exerted by viral infection in the mosquito can influence vectorial capacity (12).

The temporal dynamics of WEEV infection and associated variations in transmission have been described for four strains of *C. tarsalis* that differed in their susceptibility to WEEV (13, 14). Here, we describe varying tissue pathology associated with WEEV infection in these four strains and discuss the potential influence of these variations on vector competence.

## Materials and Methods

### Mosquito rearing and infection

Mosquito rearing and handling methods used in this study have been previously described (13). Briefly, larvae were reared at 22–24°C, with a 16 h light:8 h darkness photoperiod, and were fed ground alfalfa pellets and AquaMax^®^ (Purina Mills, LLC; St. Louis, MO, USA). Adults were maintained under a similar photoperiod at 26°C and were provided a 10% sucrose solution *ad libitum*.

Four strains of *C. tarsalis* were used in the current study: (a) WEEV resistant (WR), (b) high viremia producer (HVP), (c) Coachella Valley (COAV), and (d) Kern National Wildlife Refuge (KNWR). The WR and HVP strains were selected for refractoriness or high susceptibility, respectively, to infection with WEEV (the HVP strain was derived from the original WEEV susceptible –WS- strain) (15, 16) and have been maintained at the University of California Arbovirus Field Station since the mid 1980s. In preparation for this study, the HVP and WR strains were reselected for several generations by examining the susceptibility of single families (13). The COAV and KNWR strains were collected in California’s Riverside and Kern counties, respectively, and had been maintained as unselected colonies for 2 years prior to this study.

For viral infections, we used the WEEV strain BFS1703, which was isolated from *C. tarsalis* collected in Kern County, California, in July 1953 (17) and has been widely used for evaluating the competence of *C. tarsalis* to transmit WEEV (15, 18, 19). Virus was passaged twice in suckling mice and once in Vero cell culture prior to the beginning of the study.

The method used to infect mosquitoes has been previously described (13). Briefly, three to five day-old mated females were starved for 18 h and then allowed to engorge on viremic blood via an artificial membrane feeder (13, 20). Blood solutions contained *ca*. 3 or 5 log_10_ plaque forming units (PFU) of WEEV per 0.1 ml of chicken blood containing 14.3 freeze dried USP units of sodium heparin per milliliter (Becton-Dickson, Franklin Lakes, NJ, USA). Hereafter, these viral doses will be designated as ‘3-log’ and ‘5-log’. The 5-log dose was comparable to viremias produced by competent avian hosts that were able to infect most competent vectors, whereas the 3-log dose was similar to that produced by a less competent host, but still able to infect highly susceptible mosquito hosts such as the HVP strain. Doses below this were insufficient to infect most mosquitoes (21).

For uninfected controls, mosquitoes were allowed to feed on virus-free blood by the same method. Fully engorged females were transferred to an incubator maintained at 26°C and 18 h light:6 h darkness photoperiod, and provided with 10% sucrose solution that was changed daily.

### Immunocytochemistry

For each strain, two uninfected controls and five individuals fed on each viral dose were collected at days 1, 2, 3, 4, 7, 14, and 21 post-infectious blood meal (DPI). These mosquitoes were immobilized on wet ice, killed and fixed by injection of 10% buffered formalin (pH 7.5), and stored in 100% ETOH until further processed. Subsequently, these specimens were dehydrated, cleared, and infiltrated with paraffin as previously described (22, 23), embedded in paraffin blocks, cut into 10 μm thick serial longitudinal sections using an American Optical^®^ 820 Spencer™ microtome (American Optical Co., New York, NY, USA), mounted on microscope slides, and stored at 4°C until used for immuno-staining.

**Figure 1:**
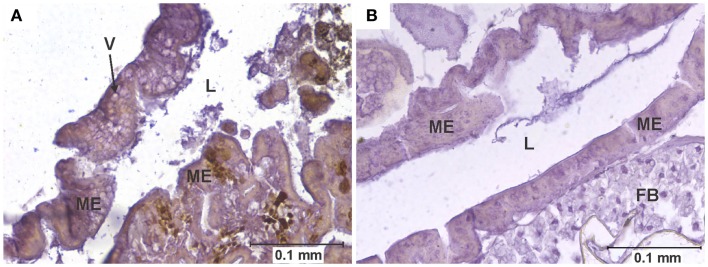
**Vacuolation of midgut epithelium**. **(A)** Section of the posterior midgut of a specimen of the COAV strain, 3 days after ingesting a blood meal containing 5-log PFU of WEEV per 0.1 ml blood. Notice the extensive formation of vacuoles in the cytoplasm. Rusty-brown staining is indicative of a positive immunocytochemical reaction, denoting the presence of WEEV antigen in the tissue. **(B)** Comparable section of the posterior midgut in an uninfected control. FB, fat body; L, midgut lumen; ME, midgut epithelium; V, vacuole.

Mounted sections were immuno-stained as previously described (14). Briefly, the avidin–biotin-peroxidase complex (ABC) technique was applied, using a 1/1,600 dilution of mouse anti-WEEV ascites fluid as the primary antibody, and the horse-anti-mouse Vectastain Elite^®^ ABC kit (Vector laboratories, Burlingame, CA, USA) for detection, following the manufacturer’s protocols. Stained sections were examined for the presence of viral antigens – as evidenced by the rusty-brown color generated by the ABC technique (Figure 1) (22)– as well as for the presence of any pathological changes, using a Nikon^®^ Optiphot™ compound microscope (Nikon Instruments Inc., Melville, NY, USA) equipped with a digital Spot RT™ camera (Diagnostic Instruments, Sterling Heights, MI, USA).

### Data analysis

Statistical analyses were performed using the SPSS^®^ software package version 13.0 for windows (SPSS Inc; Chicago, IL, USA). Chi-Square tests were used when comparing overall frequencies (i.e., time groups pooled together) between 3- and 5-log groups in each strain. If no significant differences (*P* > 0.05) between the dose groups were found within a strain, dose groups were pooled for further analysis; otherwise, each dose group was analyzed separately.

Analyses of differences among strains were performed using Kruskal–Wallis (K–W) tests because frequency data were not normally distributed for at least one strain in each one of the parameters studied. If K–W tests indicated significant (*P* < 0.05) differences among strains, *post hoc* analysis was performed by applying Chi-Square tests to all pair-wise combinations of strains. To maintain an overall alpha level of 0.05, a Bonferroni correction was applied to the *post hoc* testing.

## Results

**Figure 2:**
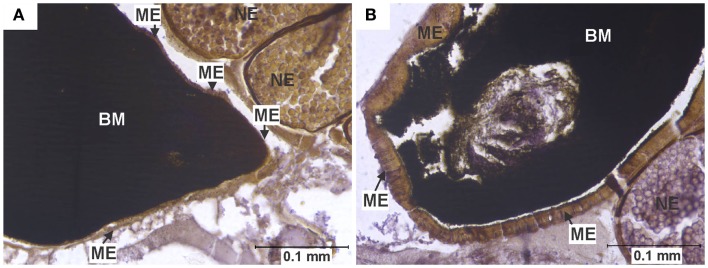
**Midgut epithelium necrosis**. **(A)** Section of the posterior midgut of a specimen of the HVP strain, 2 days after ingesting a blood meal containing 5-log PFU of WEEV per 0.1 ml blood. Notice how the epithelium has become necrotic, being reduced to a very thin band, with neither discernible cell boundaries nor traces of cytoplasm or organelles. **(B)** Comparable section of posterior midgut in an uninfected control, where no necrosis is observed. BM, blood meal (in the midgut lumen); ME, midgut epithelium; NE, non-infected egg.

**Figure 3:**
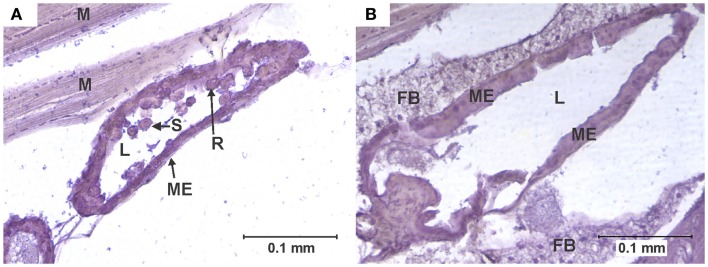
**Cell rounding and sloughing (CRS)**. **(A)** Anterior midgut section of a specimen of the KNWR strain, 14 days after ingesting a blood meal containing 3-log PFU of WEEV per 0.1 ml blood. Notice how several epithelial cells have sloughed-off into the lumen, and some rounded cells protrude from the tissue. **(B)** Comparable section of anterior midgut in an uninfected control, where no CRS is observed. FB, Fat body; L, midgut lumen; M, skeletal muscle; ME, midgut epithelium; R, rounded epithelial cell; S, sloughed epithelial cell.

**Figure 4:**
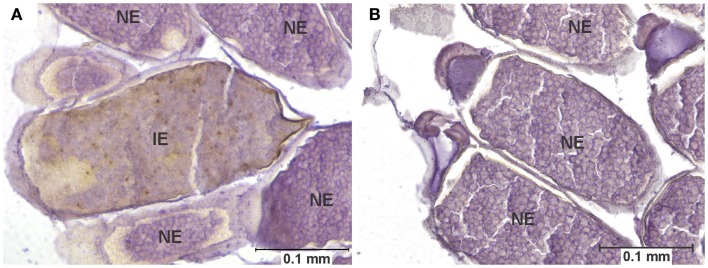
**Egg infection and pathology**. **(A)** Longitudinal section of the abdomen of specimen of the COAV strain, 21 days after ingesting a blood meal containing 5-log PFU of WEEV per 0.1 ml blood. Rusty-brown staining is indicative of a positive immunocytochemical reaction, denoting the presence of WEEV antigen in the tissue. Notice the smooth yolk texture observed in infected egg, in contrast with the uniformly granular texture of yolk observed in the neighboring uninfected eggs. **(B)** Comparable section of the abdomen of a specimen of the COAV strain, showing only normal, uninfected eggs. IE, infected egg; UE, uninfected egg.

**Figure 5:**
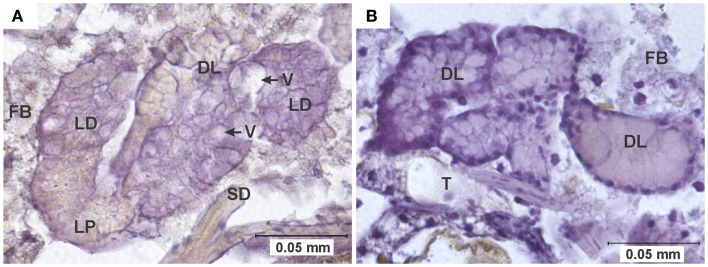
**Pathology in salivary glands**. **(A)** Salivary gland of a specimen of the HVP strain, 7 days after ingesting a blood meal containing 5-log PFU of WEEV per 0.1 ml blood. Notice the extensive cytoplasmic vacuolation of acinar cells, and the absence of staining indicative of viral antigen. **(B)** Comparable section of a normal salivary gland from an uninfected control. DL, distal lobe; FB, fat body; LD, lateral-distal lobe; LP, lateral-proximal lobe; SD, salivary duct; T, trachea; V, vacuole.

Pathological changes in infected individuals were consistently observed in the midgut epithelium (Figures 1–3) and eggs (Figure 4). Additionally, atypical cellular morphology was sporadically observed in the salivary glands of infected individuals (Figure 5); however, the consistency of salivary acini often caused them to be detached from the slides during the washes required for immunocytochemical staining, making it impossible for us to obtain consistent data across all experimental groups for this particular tissue. Therefore, only the pathological changes observed in the midgut and eggs will be further reported and discussed.

### Midgut pathology

**Figure 6:**
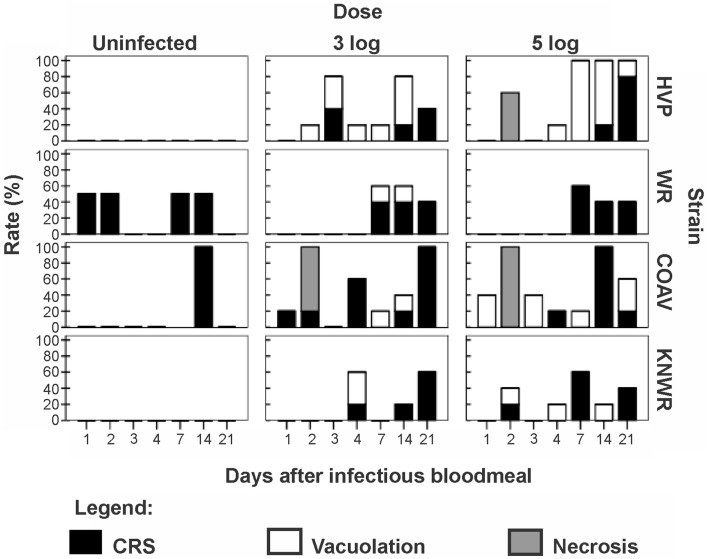
**Frequency of pathological changes in the midgut of *C*. tarsalis**. Cell rounding and sloughing was the only pathology found in uninfected individuals. Signs of necrosis were only found in HVP and COAV strains, consistently at 48 h after infection. COAV, Coachella Valley; CRS, cell rounding and sloughing; HVP, high viremia producer; KNWR, Kern National Wildlife Refuge; WR, WEEV resistant.

Three types of pathological changes were found in infected midguts: vacuolation, necrosis, and cell rounding and sloughing (CRS, Table 1 and Figure 6).

**Table 1:** **Overall frequencies of the different types of pathology found in the midgut of four different strains of *C. tarsalis* infected with WEEV**.

	HVP			WR			COAV			KNWR		
	Control (*n* = 14)	3 log (*n* = 35)	5 log (*n* = 35)	Control (*n* = 14)	3 log (*n* = 35)	5 log (*n* = 35)	Control (*n* = 10)	3 log (*n* = 35)	5 log (*n* = 34)	Control (*n* = 14)	3 log (*n* = 34)	5 log (*n* = 35)
CRS (%)	0	14	14	29	17	20	10	31	18	0	15	17
Vacuolization (%)	0	23	31	0	6	0	0	6	21	0	6	9
Necrosis (%)	0	0	9	0	0	0	0	11	15	0	0	0

*COAV, Coachella Valley; CRS, cell rounding and sloughing; HVP, high viremia producer; KNWR, Kern National Wildlife Refuge; WR, WEEV resistant*.

### Vacuolation

Arbovirus replication has been associated with the intense proliferation of intracellular vacuoles 0.3–2 μm in diameter, which are thought to be major sites of viral nucleic acid replication and virion assembly (24–26). In our study, specimens were recorded as presenting vacuolation when abundant vacuoles of the appropriate size were observed in the cytoplasm of midgut cells (Figure 1). This type of pathology was observed in all strains in the 3-log group, and in all but the WR in the 5-log group (Figure 6). In agreement with previous studies (24), no vacuolation was observed in uninfected controls.

Although vacuolation was observed as early as one DPI (COAV, 5-log group; Figure 6), there was no particular trend in the frequency of specimens showing vacuolation over time. Furthermore, no significant differences (*P* > 0.05) were found between dose groups in the overall frequency of individuals presenting vacuolation in any strain. There were, however, significant differences between strains (*X^2^* = 21.23; df = 3; *P* < 0.001), with the HVP presenting vacuolation at significantly higher frequencies than both the WR (*X*^2^ = 16.19; df = 1; *P* < 0.001) and the KNWR (*X*^2^ = 9.63; df = 1; *P* = 0.002).

### Necrosis

In close agreement with reports by Weaver et al. (6), midgut tissue necrosis was evident as a gross degeneration of cellular integrity, to the point where midgut tissue was reduced to a thin layer consisting almost exclusively of basal lamina and traces of plasma membrane (Figure 2).

In experimentally infected specimens, necrosis was found only in the HVP (5-log group) and COAV (both dose groups) strains, consistently at two DPI (Figure 6). In these strains, no significant differences (*P* > 0.05) in the frequency of specimens showing necrosis were found between dose groups. Additionally, the overall frequency of specimens showing necrosis was not significantly different between the HVP and COAV strains (*P* > 0.05).

No signs of necrosis were observed in uninfected controls of any strain.

### Cell rounding and sloughing

An individual was recorded as presenting CRS when midgut epithelial cells were observed to be either completely detached from the midgut epithelium or clearly protruding into the midgut lumen (6, 27) (Figure 3).

Among infected mosquitoes, CRS was observed in all strains and dose groups (Figure 6). Statistical analysis failed to reveal significant differences (*P* > 0.05) in the overall frequency of CRS both between dose groups within each strain, or between strains. Interestingly, CRS was also observed in uninfected controls of the WR and COAV strains (Figure 6). No significant differences (*P* > 0.05) were found between the WR and COAV strains relative to the frequency of individuals presenting CRS in infected or uninfected control groups. CRS was not seen in uninfected controls in the HVP and KNWR strains.

### Egg pathology

Eggs were interpreted as displaying pathology when all of the following characteristics were seen together: (a) positive immunocytochemical staining, indicative of the presence of viral antigen, (b) an unusually ‘smooth’ yolk texture in contrast with the granular texture of the yolk in normal eggs, and (c) distortion of the chorion (Figure 4).

**Figure 7:**
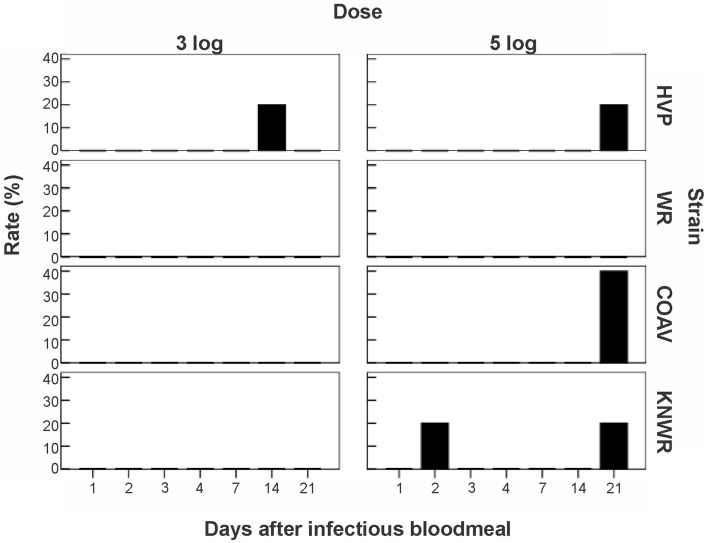
**Frequency of virus-induced pathology in eggs of *C. tarsalis***. No significant differences (*P* > 0.05) in the number of individuals presenting pathological eggs were found either between dose groups or between strains. COAV, Coachella Valley; HVP, high viremia producer; KNWR, Kern National Wildlife Refuge; WR, WEEV resistant.

Among infected mosquitoes, the HVP strain showed signs of egg pathology in both the 3- and 5-log groups. The COAV and KNWR strains showed signs of egg pathology only in the 5-log group. No evidence of egg pathology was observed in the WR strain, or in uninfected controls of any strain (Figure 7 and Table 2).

**Table 2:** **Overall frequencies of individuals presenting egg pathology associated with WEEV infection**.

	HVP		WR		COAV		KNWR	
	3 log (*n* = 35)	5 log (*n* = 35)	3 log (*n* = 35)	5 log (*n* = 35)	3 log (*n* = 35)	5 log (*n* = 34)	3 log (*n* = 34)	5 log (*n* = 35)
Frequency (%)	3	3	0	0	0	6	0	6

*COAV, Coachella Valley; HVP, high viremia producer; KNWR, Kern National Wildlife Refuge; WR, WEEV resistant*.

From a total of six females presenting egg pathology among all infected mosquitoes, five (83%) had been incubated for >14 DPI and one specimen (17%) had been incubated for two DPI (Figure 7). Statistical analysis revealed no significant differences (*P* > 0.05) between dose groups or strains in the frequency of infected individuals showing egg pathology.

## Discussion

Pathological changes were observed in the midgut, salivary glands, and eggs of WEEV-infected mosquitoes. These changes, which included CRS, vacuolation, necrosis, and egg yolk smoothing, were consistent with other reports of pathological effects of arboviruses in mosquito vectors (4, 6–9, 28) and contribute to a growing body of evidence that challenges the traditional belief that the impact of arboviral infection on mosquito cells is negligible (1–3).

The fact that CRS was observed in uninfected controls as well as infected mosquitoes is consistent with the notion proposed by Okuda et al. (27) that mosquitoes can regularly replace midgut epithelial cells following a blood meal, thus eliminating cells damaged by the toxic by-products of blood digestion. Others have suggested that viral infection triggers a high frequency of apoptosis and CRS in midgut cells, probably as a mechanism that modulates the viral load in this important tissue (6, 10, 29). Interestingly, uninfected controls of the WR strain presented the second highest frequency of CRS in our study (Table 1). Such high intrinsic turn-over rate of midgut epithelial cells may enable the WR strain to eliminate and replace infected cells before they become significant foci of viral multiplication. This mechanism could, at least in part, account for the WR strain’s refractoriness to WEEV infection.

Alternatively, CRS observed in specimens that did not receive an infective blood meal could be due to infection of our experimental strains with mosquito-specific viruses, which have been reportedly found in wild populations of *C. tarsalis* from various geographic locations (30).

Vacuolization and necrosis were observed only among individuals that received an infectious blood meal, suggesting that these types of pathological changes are closely associated with viral infection. Vacuolation has been reported as a result of arthropod-borne virus replication in infected cells (24, 25), which is consistent with our observation that the highest vacuolation rates were found in the HVP strain, and the lowest in the WR strain (Table 1 and Figure 6). Furthermore, the significantly lower vacuolation rates observed in the KNWR strain compared to the HVP strain (Table 1) suggest a lower intensity of viral replication in the former strain, and are therefore consistent with reports that found the KNWR strain to be relatively refractory to WEEV infection (13, 14). It is interesting to note that vacuolation and necrosis were in some instances observed in tissues showing no evidence of WEEV antigen presence, as indicated by the absence of immuno-staining (Figure 5). This suggests that either tissues, which had been initially infected eventually managed to clear the virus (but the pathological effect persisted), or that these tissues are infected at levels below the detection threshold of the immuno-staining methods used. Alternatively, as was the case with CRS, we cannot rule out the possibility that these pathological changes are related to the unintentional infection of our experimental strains with mosquito-specific viruses (30).

Earlier studies by Kramer et al. (31) indicated that resistance to WEEV infection in *C. tarsalis* mosquitoes of the WR strain was intimately linked to a mesenteronal barrier, because direct injection of virus into the hemocoel resulted in infection rates and viral titers comparable to those observed in highly susceptible strains. Furthermore, it has been proposed that ultrastructural alterations of the midgut, such as those caused by the ingestion of a blood meal, can be associated with increased susceptibility to viral infection (15, 32). Therefore, it is plausible that a disruption of the mesenteronal barrier caused by an infectious blood meal (for example, the midgut tissue necrosis observed in our study) would increase the odds of virus dissemination into the hemocoel, and subsequent infection of the salivary glands. Consistent with this idea, the HVP and COAV strains (which do display midgut necrosis following an infectious blood meal) have been observed to reach higher dissemination and salivary gland infection rates than the WR and the KNWR strains (14). In close agreement with observations by Weaver et al. (6), evidence of midgut necrosis disappeared by 72 h post-infectious blood meal, suggesting that this tissue has the ability to quickly recover from widespread virus-induced pathological changes.

Infected eggs displaying pathological changes were observed in three of the four strains used in this study (HVP, COAV, and KNWR) suggesting that WEEV infection in *C. tarsalis* eggs is not uncommon, even in unselected geographic strains of relatively recent colonization such as the COAV and KNWR. Interestingly, five out of six (83%) females presenting egg pathology had been incubated for at least 14 DPI, forcing them to retain eggs in their bodies for much longer than they would under natural conditions (<5 days post-blood meal). Although we cannot rule out the possibility that the egg pathology observed in these females is a response to the aforementioned forced retention of eggs, the positive immuno-staining observed in all eggs recorded as “displaying signs of pathology” indicates that they were indeed infected with WEEV. Therefore, it seems plausible that the pathological changes observed in these eggs are related to WEEV infection.

Additionally, it is worth mentioning that the only strain presenting egg infection in the low dosage (3-log) group was the HVP strain, which is characterized by developing unusually high WEEV titers (16). In contrast, the only strain that showed no egg infection was WR, which is characterized by its ability to maintain low WEEV titers (16). These observations suggest that a viral titer threshold must be reached in the infected mosquito before egg infection – and associated pathology – can occur.

Evidence of *C. tarsalis* egg infection with WEEV has been reported (33); however, subsequent field and laboratory studies have failed to produce evidence of transovarial transmission of WEEV in *C. tarsalis* (18, 34). Furthermore, although vertical transmission of WEEV in field-collected *Aedes dorsalis* has been reported once in the past (35), extensive efforts have failed to replicate this phenomenon in the laboratory (18, 36). Taken together, these data suggest that vertical transmission of WEEV in mosquitoes is a rather rare event.

In our study, all eggs found to be positive for WEEV antigen presented clear signs of pathological changes in their yolk and chorion, which probably rendered these eggs non-viable. Therefore, our data support the notion that *C. tarsalis* does not normally transmit WEEV transovarially (18, 34). Nevertheless, these infected eggs were surrounded by apparently healthy, viable eggs (Figure 4), suggesting that they could be oviposited as part of otherwise normal egg rafts. This sporadic occurrence of non-viable, virus-laden eggs may explain why Thomas (33) was able to isolate virus from *C. tarsalis* egg rafts deposited by orally infected females, but could not conclusively demonstrate transovarial transmission of WEEV. Interestingly, a similar phenomenon has been observed in *C. tarsalis* infected with West Nile virus (WNV); although egg infection is frequent, trans-generational transmission occurs only rarely (W.K. Reisen, personal communication).

Romoser et al. (28), referring to the infection of *A. mcintoshi* eggs with Rift Valley fever virus, hypothesized that the oviposition of virus-laden eggs might have important epidemiological consequences, as it represents a mechanism by which viral particles could be deposited directly in the aquatic environment inhabited by mosquito larvae, which could eventually ingest these virions. The ingestion of infective viral particles during larval stages has been reported to result in transstadially transmitted infections, producing adult mosquitoes that are able to transmit virus to new hosts when they blood feed (37). Our observations of WEEV infection in *C. tarsalis* eggs are consistent with the hypothesis proposed by Romoser and his collaborators, and suggest that this process could take place in at least some mosquito/virus systems, therefore potentially playing a role in the environmental persistence of vector-borne viruses.

As noted in the results, difficulties in salivary gland tissue preparation and immuno-staining precluded a systematic assessment of salivary gland pathology at this time. However, several cases of atypical cellular morphology (vacuolation) were observed in the salivary glands of infected individuals (Figure 5). Salivary gland pathology associated with arbovirus infection has been found in other studies (7–9, 28, 38). Girard et al. (8, 9) have suggested that WNV-induced damage to either salivary glands or ganglia controlling salivation in *Culex pipiens and Culex quinquefasciatus* might result in reduced volumes of saliva being expectorated, which would in turn cause a reduction in feeding efficiency and the viral load injected into new hosts. Furthermore, it has been reported that long-term arboviral infections result in progressive declines in transmission rates and/or the volume of virus expectorated by infected mosquitoes (9, 13, 19). Although the instances of salivary gland pathology we observed in this study are consistent with the idea of the progressive decline of transmission rates due to damage to salivary cells, more research is needed to establish the exact role of salivary gland pathology in vector competence.

## Author Contributions

Marco V. Neira, Farida Mahmood, William K. Reisen, Calvin B. L. James, and William S. Romoser designed the experiments. Farida Mahmood reared mosquitoes, performed experimental infections and fixed specimens. Marco V. Neira and William S. Romoser performed immunocytochemical staining, microscopical analysis, data analysis, and wrote the manuscript. All authors reviewed, edited and approved the manuscript.

## Conflict of Interest Statement

The authors declare that the research was conducted in the absence of any commercial or financial relationships that could be construed as a potential conflict of interest.

## References

[1] Chamberlain RW Sudia WD Mechanism of transmission of viruses by mosquitoes. Annu Rev Entomol (1961) 6:371–90 10.1146/annurev.en.06.010161.002103 13692218

[2] McLintock J Mosquito-virus relationships of American encephalitides. Annu Rev Entomol (1978) 23:17–37 10.1146/annurev.en.23.010178.000313 24407

[3] Hardy JL Houk EJ Kramer LD Reeves WC Intrinsic factors affecting vector competence of mosquitoes for arboviruses. Annu Rev Entomol (1983) 28:229–62 10.1146/annurev.en.28.010183.001305 6131642

[4] Mims CA Day MF Marshall ID Cytopathic effect of Semliki forest virus in the mosquito *Aedes aegypti*. Am J Trop Med Hyg (1966) 15:775–84 5917634 10.4269/ajtmh.1966.15.775

[5] Weaver SC Scott TW Lorenz LH Lerdthusnee K Romoser WS Togavirus-associated pathologic changes in the midgut of a natural mosquito vector. J Virol (1988) 62:2083–90 2896802 10.1128/jvi.62.6.2083-2090.1988 PMC253299

[6] Weaver SC Lorenz LH Scott TW Pathologic changes in the midgut of *Culex tarsalis* following infection with western equine encephalomyelitis virus. Am J Trop Med Hyg (1992) 47:691–701 1449210 10.4269/ajtmh.1992.47.691

[7] Bowers DF Coleman CG Brown DT Sindbis virus-associated pathology in *Aedes albopictus* (Diptera: Culicidae). J Med Entomol (2003) 40:698–705 10.1603/0022-2585-40.5.698 14596286

[8] Girard YA Popov V Wen J Han V Higgs S Ultrastructural study of West Nile virus pathogenesis in *Culex pipiens quinquefasciatus* (Diptera: Culicidae). J Med Entomol (2005) 42:429–44 10.1603/0022-2585(2005)042[0429:USOWNV]2.0.CO;2 15962797

[9] Girard YA Schneider BS McGee CE Wen J Han VC Popov V Salivary gland morphology and virus transmission during long-term cytopathologic West Nile virus infection in *Culex* mosquitoes. Am J Trop Med Hyg (2007) 76:118–28 Available from: http://www.ajtmh.org/content/76/1/118.full 17255239

[10] Vaidyanathan R Scott TW Apoptosis in mosquito midgut epithelia associated with West Nile virus infection. Apoptosis (2006) 11:1643–51 10.1007/s10495-006-8783-y 16820968

[11] Karpf AR Brown DT Comparison of Sindbis virus-induced pathology in mosquito and vertebrate cell cultures. Virology (1998) 240:193–201 10.1006/viro.1997.8914 9454692

[12] Ciota AT Kramer LD Vector-virus interactions and transmission dynamics of West Nile virus. Viruses (2013) 5:3021–47 10.3390/v5123021 24351794 PMC3967159

[13] Mahmood F Chiles RE Fang Y Green EN Reisen WK Effects of time after infection, mosquito genotype, and infectious viral dose on the dynamics of *Culex tarsalis* vector competence for western equine encephalomyelitis virus. J Am Mosq Control Assoc (2006) 22:272–81 10.2987/8756-971X(2006)22[272:EOTAIM]2.0.CO;2 17019773

[14] Neira Oviedo MV Romoser WS James CB Mahmood F Reisen WK Infection dynamics of western equine encephalomyelitis virus (Togaviridae: Alphavirus) in four strains of *Culex tarsalis* (Diptera: Culicidae): an immunocytochemical study. Res Rep Trop Med (2011) 2011:65–77 10.2147/RRTM.S13946 22629118 PMC3358002

[15] Hardy JL Apperson G Asman SM Reeves WC Selection of a strain of *Culex tarsalis* highly resistant to infection following ingestion of western equine encephalomyelitis virus. Am J Trop Med Hyg (1978) 27:313–21 646024 10.4269/ajtmh.1978.27.313

[16] Hardy JL Reeves WC Bruen JP Presser SB Vector competence of *Culex tarsalis* and other mosquito species for western equine encephalomyelitis virus. In: Kurstak E editor. Arctic and Tropical Arboviruses. New York: Academic Press Inc (1979). p. 157–71

[17] Reeves WC Hammon WM Longshore WA Jr. Mc CH Geib AF Epidemiology of the arthropod-borne viral encephalitides in Kern County, California, 1943-1952. Publ Public Health Univ Calif (1962) 4:1–257 14491029

[18] Hardy JL Reeves WC Experimental studies in infection in vectors. In: Reeves WC editor. Epidemiology and Control of Mosquito-Borne Arboviruses in California, 1943–1987. Sacramento, CA: California Mosquito Vector Control Association (1990). p. 145–253

[19] Reisen WK Meyer RP Presser SB Hardy JL Effect of temperature on the transmission of western equine encephalomyelitis and St. Louis encephalitis viruses by *Culex tarsalis* (Diptera: Culicidae). J Med Entomol (1993) 30:151–60 8433322 10.1093/jmedent/30.1.151

[20] Rutledge LC Ward RA Gould DJ Studies on the feeding response of mosquitoes to nutritive solutions in a new membrane feeder. Mosq News (1964) 24:407–19

[21] Reisen WK Chiles RE Martinez VM Fang Y Green EN Experimental infection of California birds with western equine encephalomyelitis and St. Louis encephalitis viruses. J Med Entomol (2003) 40:968–82 10.1603/0022-2585-40.6.968 14765678

[22] Faran ME Romoser WS Routier RG Bailey CL Use of the avidin–biotin-peroxidase complex immunocytochemical procedure for detection of Rift valley fever virus in paraffin sections of mosquitoes. Am J Trop Med Hyg (1986) 35:1061–7 3532843 10.4269/ajtmh.1986.35.1061

[23] Leon R The localization of Venezuelan equine encephalitis virus in *Aedes taeniorhynchus* mosquitoes using nucleic acid hybridization and immunocytochemistry. Doctoral Dissertation. Athens, OH: Ohio University (2000). 382 p.

[24] Grimley PM Berezesky IK Friedman RM Cytoplasmic structures associated with an arbovirus infection: loci of viral ribonucleic acid synthesis. J Virol (1968) 2:1326–38 5750316 10.1128/jvi.2.11.1326-1338.1968 PMC375472

[25] Virtanen I Wartiovaara J Virus-induced cytoplasmic membrane structures associated with Semliki forest virus infection studied by the freeze-etching method. J Virol (1974) 13:222–5 4129842 10.1128/jvi.13.1.222-225.1974 PMC355278

[26] Ishikawa T Konishi E Mosquito cells infected with Japanese encephalitis virus release slowly-sedimenting hemagglutinin particles in association with intracellular formation of smooth membrane structures. Microbiol Immunol (2006) 50:211–23 10.1111/j.1348-0421.2006.tb03788.x 16547419

[27] Okuda K de Almeida F Mortara RA Krieger H Marinotti O Bijovsky AT Cell death and regeneration in the midgut of the mosquito, *Culex quinquefasciatus*. J Insect Physiol (2007) 53:1307–15 10.1016/j.jinsphys.2007.07.005 17716685

[28] Romoser WS Neira Oviedo M Lerdthusnee K Patrican LA Turell MJ Dohm DJ Rift valley fever virus-infected mosquito ova and associated pathology: possible implications for endemic maintenance. Res Rep Trop Med (2011) 2:121–7 10.2147/RRTM.S13947 PMC6415639 30881185

[29] Kramer LD Hardy JL Presser SB Houk EJ Dissemination barriers for western equine encephalomyelitis virus in *Culex tarsalis* infected after ingestion of low viral doses. Am J Trop Med Hyg (1981) 30:190–7 7212166 10.4269/ajtmh.1981.30.190

[30] Tyler S Bolling BG Blair CD Brault AC Pabbaraju K Armijos MV Distribution and phylogenetic comparisons of a novel mosquito flavivirus sequence present in *Culex tarsalis* mosquitoes from western Canada with viruses isolated in California and Colorado. Am J Trop Med Hyg (2011) 85:162–8 10.4269/ajtmh.2011.10-0469 21734143 PMC3122362

[31] Kramer LD Hardy JL Houk EJ Presser SB Characterization of the mesenteronal infection with Western equine encephalomyelitis virus in an incompetent strain of *Culex tarsalis*. Am J Trop Med Hyg (1989) 41:241–50 2774065 10.4269/ajtmh.1989.41.241

[32] Houk EJ Midgut ultrastructure of *Culex tarsalis* (Diptera: Culcidae) before and after a bloodmeal. Tissue Cell (1977) 9:103–18 10.1016/0040-8166(77)90052-0 898169

[33] Thomas LA Distribution of the virus of western equine encephalomyelitis in the mosquito vector, *Culex tarsalis*. Am J Hyg (1963) 78:150–65 14063719 10.1093/oxfordjournals.aje.a120334

[34] Chamberlain RW Sudia WD The North American arthropod-borne encephalitis viruses in *Culex tarsalis* Coquillett. Am J Hyg (1957) 66:151–9 13458180 10.1093/oxfordjournals.aje.a119892

[35] Fulhorst CF Hardy JL Eldridge BF Presser SB Reeves WC Natural vertical transmission of western equine encephalomyelitis virus in mosquitoes. Science (1994) 263:676–8 10.1126/science.8303276 8303276

[36] Kramer LD Reisen WK Chiles RE Vector competence of *Aedes dorsalis* (Diptera: Culicidae) from Morro Bay, California, for western equine encephalomyelitis virus. J Med Entomol (1998) 35:1020–4 9835696 10.1093/jmedent/35.6.1020

[37] Turell MJ Linthicum KJ Beaman JR Transmission of Rift valley fever virus by adult mosquitoes after ingestion of virus as larvae. Am J Trop Med Hyg (1990) 43:677–80 2267972 10.4269/ajtmh.1990.43.677

[38] Lam KS Marshall ID Dual infections of *Aedes aegypti* with arboviruses. II. Salivary-gland damage by Semliki forest virus in relation to dual infections. Am J Trop Med Hyg (1968) 17:637–44 5672794

